# Mouse and human embryonic genome activation initiate at the one-cell stage

**DOI:** 10.3389/fcell.2025.1594995

**Published:** 2025-07-30

**Authors:** Maki Asami, Anthony C. F. Perry

**Affiliations:** Laboratory of Mammalian Molecular Embryology, Department of Life Sciences, University of Bath, Bath, United Kingdom

**Keywords:** transcription, fertilization, one-cell embryo, embryonic genome activation (EGA), immediate EGA, zygotic genome activation (ZGA), embryonic genome repression (EGR), single-cell RNA-sequencing

## Abstract

At the moment of their union, fertilizing gametes (sperm and oocyte) are transcriptionally silent: gene expression has to be initiated within the resulting embryo, a process termed embryonic genome activation, EGA. Until recently, EGA was believed to occur at the two-cell stage (mouse) or four-to-eight-cell stage (human), but new evidence from single-cell RNA-sequencing (scRNAseq) suggests that it initiates at the one-cell stage in both species. Precise time-course scRNA-seq of mouse one-cell embryos revealed an EGA program referred to as immediate EGA, iEGA: iEGA occurred from within 4 h of fertilization, mainly from the maternal genome, with paternal genomic transcription from ∼10 h. Significant low-magnitude upregulation similarly occurred in healthy human one-cell embryos. In both species, new transcripts were canonically spliced, and expression predicted embryonic processes and regulatory transcription factors (TFs) associated with cancer, including MYC/c-Myc. Blocking their activities in mouse one-cell embryos induced acute developmental arrest and disrupted iEGA. Inhibiting c-Myc induced upregulation of hundreds of genes, implying that they are normatively repressed, a phenomenon we term embryonic genome repression, EGR. iEGA is downregulated coincidentally with a subsequent, higher-amplitude wave of gene expression (referred to as ‘major EGA’ or ‘major ZGA’) in two-cell (mouse) or 4–8-cell (human) embryos. We suggest that iEGA is continuous with gene expression previously termed ‘minor EGA’ (or ‘minor ZGA’) and that the regulation of iEGA and major EGA are distinctive. The pattern of gene upregulation in iEGA illuminates processes involved at the onset of development, with implications for epigenetic inheritance, stem cell-derived embryos and cancer.

## 1 Introduction

When a sperm and an oocyte (egg) combine in fertilization, they are transcriptionally silent (Balhorn, 1982; Zuccotti et al., 1995). Transcription must therefore be initiated on the newly-formed embryonic genome, a process generically referred to as embryonic genome activation, EGA. This Perspective considers how EGA is initiated in mouse and human embryos, with implicit relevance to other mammalian species. A central tenet is that mouse and human EGA begin in one-cell embryos during fertilization.

**FIGURE 1:**
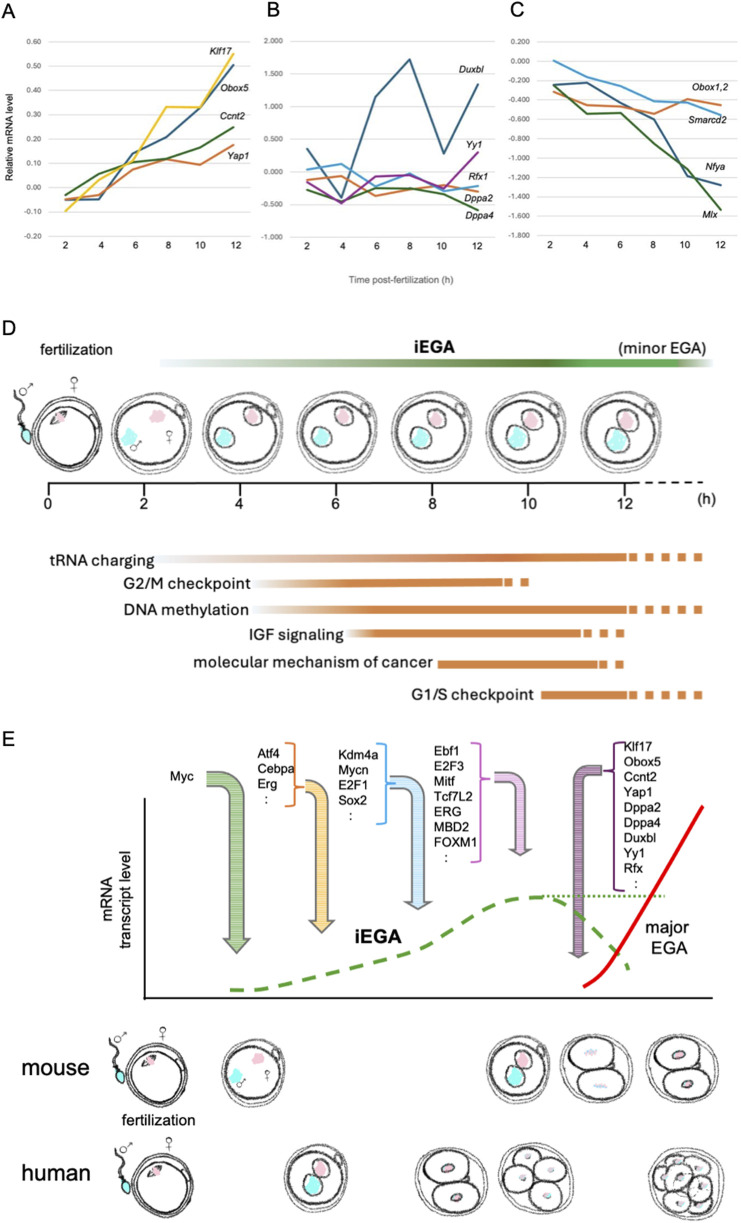
Expression profiles of candidate major EGA activating TF mRNAs during iEGA. **(A)** *Klf17*, *Obox5*, *Ccnt2* and *Yap1* are iEGA genes whose levels increase (Asami et al., 2023). Y-axis: log2FC. **(B)** Other predicted major EGA TF mRNA levels do not change, or **(C)** decrease. For all analyses, FDR<5%. **(D)** The succession of pathways predicted by iEGA (FDR<5%) include tRNA charging (e.g., EARS2, HARS), G2/M checkpoint-associated genes (e.g., CHEK1, CKS1B), DNA methylation-associated genes (e.g., HIST1H4A, SAP30), IGF signaling-associated genes (e.g., CSNK2A1, IGFBP4), genes associated with the molecular mechanism of cancer (e.g., AKT1, WNT4) and G1/S checkpoint-associated genes (e.g., CCNE2, TP53). **(E)** Predicted mouse and human gene activators in iEGA and major EGA (distinct waves of transcription). Mouse and human iEGA profiles overlap. Transcription regulators were inferred by IPA of mouse iEGA (FDR<5%) at 4, 6, 8 and 12 h, and are indicated where they are first predicted to act.

Fertilization describes the period linking sperm-oocyte fusion to chromosome mingling just prior to the first mitotic cytokinesis: the gamete-to-embryo transition (see Box 1 for a glossary of terms) (Yanagimachi, 1994). In the mouse, fertilization takes around 16 h, and in humans a little longer (Suzuki et al., 2016) (Figure 1). The product is a presumptively totipotent cell capable of engendering the full-term development of an individual (Condic, 2014) (Box 1). The emergence of totipotency during fertilization coincides with multiple integrated dynamic processes, including meiotic progression (Yanagimachi, 1994), signalling fluxes that involve calcium oscillations (Cuthbertson et al., 1981) and phospho-relays (Perry and Verlhac, 2008), transmission (to the embryo) and activation of maternal factors (including protein and RNAs carried over from the oocyte following sperm union; Wu and Dean, 2020), the onset of maternal transcript (and other maternal factor) degradation (Clegg and Piko, 1983), extensive and parent-specific chromatin remodeling including genome demethylation (Adenot et al., 1997; Liu et al., 2014; Mayer et al., 2000), pronucleus formation (Yanagimachi, 1994), and a program of intracellular force changes involving surges during chromatin remodelling and cytokinesis (Duch et al., 2020). Although little is known about them, additional changes, including organelle reconfiguration (e.g., migration and restructuring), macromolecular trafficking and phase separation (Hyman et al., 2014; Ling et al., 2022), are likely to play formative roles, not least because in cellular terms, mouse and human one-cell embryos are relatively large (≥170 pL, compared to ∼4 pL for a typical somatic cell). Studying the emergence of totipotency is made more challenging by the likelihood of functional redundancy and complementarity during fertilization. For example, phospholipase C-zeta, which is the oocyte-activating trigger for embryogenesis delivered by a fertilizing sperm, is dispensable for developmental activation (Hachem et al., 2017). Calcium ion mobilization during mammalian fertilization presumptively activates phospho-signalling but is dispensable for full-term development (Suzuki et al., 2010). Compensation may also confound the analysis of transcriptional regulation, producing multiple, occasionally incompatible, inferred mechanisms (Gassler et al., 2022; Ji et al., 2023).

BOX 1 **Glossary of selected terms used.**

**Table:** 

Term	Definition
Fertilization	Period linking gamete fusion to parental chromosome mingling (syngamy) following pronuclear membrane breakdown (Yanagimachi, 1994)
Minor EGA, minor ZGA	Transcription in late (defined without good temporal resolution) one-cell embryos (Hamatani et al., 2004; Xue et al., 2013): approximately, S-phase of one-cell to G1-phase of two-cell stages.
Major EGA, major ZGA	Transcription in two-cell (mouse) or four-to-eight-cell (human) embryos (Braude et al., 1988; Hamatani et al., 2004; Xue et al., 2013).
Plenipotent	Able to give rise to any embryonically-derived cell type (Condic, 2014).
Pluripotent	Able to give rise to any embryonically-derived cell type present in the embryo proper
Totipotent	Cell that is normatively able to give rise to an entire individual (Condic, 2014). In the mouse, only two cell types are totipotent: one-cell embryos and the blastomeres of a two-cell embryo (Katayama et al., 2010; Rossant, 1976; Tarkowski, 1959). Defining ‘totipotency’ to include cells that can give rise to all cell types does not capture additional tiers of information necessary to choreograph full development. Cells that can normatively give rise to all cell types but not offspring have been labelled plenipotent (Condic, 2014).
Zygote	One-cell embryo
Zygotic genome activation, ZGA	ZGA has been used synonymously with EGA for historical reasons, but is inappropriate when describing processes that occur in two-, four- or eight-cell embryos (e.g., major ZGA), because they do not occur in zygotes.

Because these processes reflect and determine the intracellular milieu during fertilization, understanding them should lead to better models of totipotency establishment, maintenance and exit. This is challenging because they are complex, occur at small scale in low numbers of transient cell types (e.g., one-cell embryos) and are integrative, thwarting reductionist approaches. Totipotency is a transitory state and totipotent stem cells do not exist. Parallels have been drawn between the two-cell embryonic state and a sub-population within pluripotent stem cell (PSC) cultures, referred to as two-cell-like cells in the mouse (Macfarlan et al., 2012); a corresponding state has been identified between eight-cell embryos and eight-cell-like PSCs in humans (Mazid et al., 2022). Such parallels may indicate overlaps between the mechanisms that regulate pluripotency and totipotency, but the embryos and stem cells in which they occur are respectively distinct transcriptionally, structurally, metabolically, morphologically and developmentally.

The onset of EGA provides a read-out of the processes controlling totipotency: it is a response to cellular events in the nascent one-cell embryo that are critical for the emergence of the totipotent state. We refer to the initiation of EGA in the first 12 h after fertilization in the mouse, as immediate EGA, iEGA, and argue that an analogous process occurs in human one-cell embryos (Asami et al., 2022; Asami et al., 2023). Gene expression has previously been reported in human and mouse late one-cell embryos, termed ‘minor EGA’ (‘minor ZGA’; Box 1) in the mouse, but it has not been defined with temporal precision and its biological role has not been acknowledged (Abe et al., 2015; Hamatani et al., 2004; Park et al., 2013; Wang et al., 2004; Xue et al., 2013; Zeng and Schultz, 2005). We argue that minor EGA is a continuation of iEGA (Asami et al., 2023). iEGA is followed by ‘major EGA’ (‘major ZGA’; Box 1) in mouse two-cell embryos and at the four-to-eight-cell stage in humans (Hamatani et al., 2004; Park et al., 2013; Wang et al., 2004; Xue et al., 2013; Zeng and Schultz, 2005) (Figure 1).

We now consider iEGA in the context of major EGA and preparation for preimplantation development. We also introduce the notion of embryonic genome repression, EGR, which corresponds to a specific profile of transcriptional repression identified in the mouse during iEGA.

## 2 The onset of embryonic transcription: iEGA

Profiling the onset of embryonic transcription has proven elusive. Studies have relied on embryos derived *in vivo* for which the time of fertilization was indeterminate, even though, in the mouse, oocytes are fertilizable for >12 h post-ovulation (Marston and Chang, 1964), the time of coitus and duration of sperm passage and fusion at the fertilization site varies (Suarez, 1987), and one-cell embryo morphology and time since fertilization are not reliably correlated (Adenot et al., 1997). Some studies (Table 1) used hundreds or thousands of embryos (Ko et al., 2000; Abe et al., 2015; Hamatani et al., 2004; Park et al., 2013; Wang et al., 2004), potentially smoothing signals (Olsen and Baryawno, 2018) and precluding the degree of inter-embryo synchrony necessary for accurate one-cell embryo transcriptome profiling. In most cases, transcripts have been isolated by poly(A) capture, but the length of poly(A) tails is controlled in early embryos as a means to regulate translation, potentially skewing outputs such that they reflect mRNA polyadenylation in addition to *de novo* gene expression (Blower et al., 2013; Daniels et al., 1995; Hamatani et al., 2004; Temeles and Schultz, 1997). Additionally, maternally-derived mRNA in one-cell embryos (Qiao et al., 2020) may compromise the detection of significant, low-amplitude embryonic gene expression.

**TABLE 1:** Summary of mouse and human datasets containing one- and two-cell embryo transcriptomes.

Species	Data type	References	Accession no(s)	Embryo preparation	Embryo stages	Library preparation method	Cell numbers	Cut-off
Mouse	3′-EST sequences	Ko et al. (2000)	C75935-C81630, C85044-C88357, AU014577-AU024803, AU040095-AU046300	Natural mating	mII to blastocyst	Total RNA-derived PCR-based cDNA library construction	>1,528 x mIl, 1,137 × 1C, 397 × 2C, 32 × 4C, 230 × 8C, 42 × 16C, 40 x blastocyst	na
	Microarray	Hamatani et al. (2004)	GSE936	Natural mating	mII to blastocyst	Quickprep micro poly-A RNA Extraction Kit	500 x mII, 500 × 1C, 500 × 2C, 500 × 4C, 500 × 8C, 500 x morula, 500 x blastocyst	FDR <1%
	Microarray	Wang et al. (2004)	(Deposited Arrayexpress)	Natural mating (defined by phCG)	GV to blastocyst	Total RNA derived cDNA synthesis	<60 x GV, <55 x mII, <70 × 1C, <134 × 2C, <131 × 4C, <95 × 8C, <42 × 8C, <70 x blastocyst	na
	Affymetrix MOE430 microarray	Zeng and Schultz (2005)	Not given	Natural mating	mII, 1C and 2C	cRNA preparation according to the Affymetrix Small Sample Prep Technical Bulletin	Pools of ∼325 eggs; 335 1C embryos; 380 2-cell embryos	FDR <5%
	RNA-sequencing	Park et al. (2013)	DRA001066	IVF	mII to 4C	Total RNA-seq libraries: SOLiD Total RNA-seq kit	10,000 cells from each stage	FDR <5%
	Single-cell RNA-sequencing	Xue et al. (2013)	GSE44183	Natural mating (defined by hCG)	mII to morula	Tang et al., 2010 (Illumina)	Single cell	FDR <5%
	RNA-sequencing	Deng et al., 2014	GSE45719	Natural mating (defined by hCG)	mII to blastocyst	Poly(A) RNA-seq libraries, Smart-seq2 (Takara Clontech)	Single cell	na
	RNA-sequencing	Abe et al. (2015)	not given	IVF	mII to blastocyst	Total RNA RNA-Seq libraries, mRNA-seq Sample Preparation Kit (Illumina)	3,000 x mII, 3,000 × 1C, 4,500 × 2C, 2,800 x 4C, 1,400 x morula, and 700 x blastocysts	na
	Single-cell RNA-sequencing	Fan et al. (2015)	GSE53386	Natural mating	mII to blastocyst	SUPeR-seq	Single cell	p-value <0.05
	RNA-sequencing	Abe et al. (2018)	DRA006557	IVF	mII and 2C	Total RNA RNA-Seq libraries, mRNA-Seq Sample Preparation Kit (Illumina)	4,500 embryos	na
	Smart-seq2 long-read RNA-sequencing	Qiao et al. (2020)	GSE138760	Natural mating	mII to blastocyst	Total RNA cDNA amplified via the Smart-seq2 protocol	Pools of 150 oocytes; 150 × 1C; 100 × 2C; 50 × 4C; 25 × 8C; 20 × 32-64C blastocyst	unknown
	RNA-sequencing	Zhang et al. (2022)	GSE169632	IVF	mII to blastocyst	Total RNA-seq libraries: SMART-Seq Stranded Kit (Takara Clontech) Poly(A) RNA-seq libraries: SMARTer ultralow input RNA cDNA preparation kit (Takara Clontech)	100 to 250 oocytes or embryos	FDR <1%
	Single-cell RNA-sequence and DNA microarray	Asami et al. (2023)	GSE222130, GSE64648, GSE64649 and GSE64650	ICSI	mII and 1C (2-, 4-, 6-, 8-, 10-, 12-hpf)	SMARTer Stranded Total RNA-Seq Kit v1 and 2 – Pico Input Mammalian (Takara Clontech)	Single cell	FDR <5%
Human	Microarray	Vassena et al. (2011)	GSE29397	Not stated	mII to blastocyst	Affymetrix Human Gene 1.0 ST array	Not stated	p-value <0.05
	Single-cell RNA-sequencing	Yan et al. (2013)	GSE36552	IVF	mII to blastocyst	Step-by-step single-cell RNA-seq TrueSeq DNA library preparation kit (Illumina)	Single cell	p-value <0.01
	Single-cell RNA-sequencing	Xue et al. (2013)	GSE44183	ICSI	mII to morula	Tang et al., 2010 (Illumina)	Single cell	FDR <5%
	Single-cell RNA-sequencing	Leng et al. (2019)	GSE133856	IVF	mII to morula	Total RNA cDNA amplified via the Smart-seq2 protocol	Single cell	p-value <0.05
	Single-cell RNA-sequencing	Asami et al. (2022)	GSE157834	ICSI	mII and 1C	Clontech SMARTer Total RNA-Seq Kit Pico Input (V2) system (Takara Clontech)	Single cell	FDR <5%

Recent high-resolution, polyadenylation-independent scRNA-seq time-course profiling of precisely-staged mouse one-cell embryos has addressed several of these caveats (Asami et al., 2023). Embryo synchrony within 5 min per time-point was achieved by coordinated microinjection and, following scRNA-seq of embryos at different points on the resulting time-course, revealed a program of embryonic gene expression initiating within 4 h of fertilization (Asami et al., 2023). Using a false discovery rate (FDR) of <5%, 1,777 genes were found to be upregulated in iEGA (i.e., within the first 12 h of sperm-egg union) compared to mature, fertilizable metaphase II (mII) oocytes. Of these, ∼90% were predicted to be RNA polymerase II- (PolII-) generated transcripts that were canonically-spliced, with transcription predominantly from the maternal genome (Asami et al., 2023). Analogous time-course profiling in human embryos is impracticable, but scRNA-seq of imprecisely-staged, apparently healthy (e.g., bipronuclear; 2PN) human one-cell embryos also revealed significant (FDR <5%) transcriptional upregulation. At 12.7% (FDR<5%), the overlap between mouse iEGA and presumptive iEGA in human one-cell embryos (henceforth referred to as iEGA) was modest, consistent with human embryo genetic heterogeneity (e.g., they were from different ethnicities), lack of synchrony, and the precise time of fertilization being unknown, any of which might conceal human-mouse similarities. However, pathways predicted for mouse and human iEGA overlapped, with the human dataset most closely corresponding to an early (4 h) timepoint in the mouse time-course (Asami et al., 2022). Mouse iEGA pathway terms included cell cycle regulation (e.g., the meiotic-to-mitotic cell cycle transition), metabolism (e.g., IGF signalling) and DNA methylation (e.g., transcriptional repression signalling) (Mayer et al., 2000; Wossidlo et al., 2011; Yoshida et al., 2007). Many analogous pathway terms are represented in human iEGA (Asami et al., 2022) (Figure 1). Consistent with a previous report of murine endogenous retrovirus (MuERV) expression in one-cell embryos (Kigami et al., 2003), *LTR*, *Pol* and *Gag* genes (Rowe and Trono, 2011) were upregulated at hundreds of loci from 8 h, showing that MuERV gene activation is a feature of iEGA (Asami et al., 2023). Upregulated genes in human one-cell embryos also included 63 endogenous retrovirus (*hERV*) loci (Asami et al., 2022).

## 3 Regulation of iEGA

What might we infer from iEGA about upstream and downstream transcription regulators in one-cell embryos? The putative EGA regulator gene, *Dux* (Hendrickson et al., 2017; De Iaco et al., 2017), is upregulated in mouse iEGA (Asami et al., 2023). Dux has been shown to recruit p300/CBP to regulatory regions in minor EGA genes including *Obox4*, *Zscan4s* and *Usp17s*, independently of p300/CBP catalytic acetylation (Xiao et al., 2025); recruitment of p300/CBP may also be primed by H3.3S31ph (Zhang et al., 2025). This may help facilitate PolII localization and the transition from minor to major EGA (Xiao et al., 2025). However, although genetic deletion experiments show that Dux promotes early development in the mouse (possibly by regulating the cell cycle), not all Dux target genes are dysregulated in *Dux*-null embryos, some of which complete full-term development (De Iaco et al., 2020). Moreover, Dux-responsive genes were not upregulated in iEGA, suggesting that iEGA is independent of Dux and its paralogs (Asami et al., 2023). In humans, *DUX4* (the mouse *Dux* ortholog) (Hendrickson et al., 2017) and other TFs postulated to drive human cleavage-stage (major) EGA, including *OCT4* (Gao et al., 2018) and *LEUTX* (Jouhilahti et al., 2016), were absent from iEGA (Asami et al., 2022).

Many transcription activators predicted by both mouse and human iEGA (FDR<5%) were oncogenes, including (with corresponding mouse species orthologs), MYC, MYCN, RABL6, FYN and E2F4 (Asami et al., 2022; 2023). In the mouse, *trans*-activation by c-Myc, Mycn, Erg and Atf4, whose human counterparts have well-documented roles in cancer (Baluapuri et al., 2020; Papas et al., 1990; Shen-Li et al., 2000; Wortel et al., 2017), was predicted to have occurred within 8 h of fertilization (Asami et al., 2023). Each of these proteins was present in mouse mII oocytes and one-cell embryos, although corresponding mRNAs were often undetectable: c-Myc was present in immature oocytes and localized to spindles in mII oocytes (Alexandrova et al., 1995; Asami et al., 2023; González-Prieto et al., 2015). RNA-seq following high-sensitivity assay for transposase-accessible chromatin (ATAC-seq) of human one-cell embryos (albeit abnormal, 3PN) revealed regions of open chromatin that were distal to major EGA regulatory regions, enriched for transcription factor-binding sites and overlapping with DNA hypomethylated domains (Wu et al., 2018). Many of these distal regions become inaccessible after major EGA in a transcription-dependent manner. Such chromatin dynamics are conserved in mice.

Both c-Myc, and its canonical heterodimeric co-activating partner, Max, were present in oocytes and one-, two- and four-cell embryos (Asami et al., 2023). The c-Myc cleavage product, Myc-nick (Conacci-Sorrell et al., 2010) was present in one-cell embryos, and other isoforms became readily detectable in cleavage-stage embryos, indicative of dynamic c-Myc regulation during and after iEGA. Treating mouse one-cell embryos with structurally distinct inhibitors of c-Myc-Max heterodimerization (MYCi975 and 10058-F4) that block c-Myc gene-regulatory activity (Han et al., 2019; Huang et al., 2006) induced embryo developmental arrest at one- or two-cell stages (Asami et al., 2023). scRNA-seq following 10058-F4 treatment identified 577 genes expressed at reduced levels compared to untreated controls (FDR<5%), including 95.4% of the differentially expressed genes that overlapped with iEGA genes. The list contained known c-Myc targets and predicted involvement (*p* < 0.01) in G2/M DNA damage regulation, cell-cycle control of chromosome replication and nucleotide excision repair. Perhaps c-Myc potentiates iEGA by poised transcription complex formation (Rahl et al., 2010) in mII oocytes and modulates transcription akin to its amplification of gene expression in cancer (Lin et al., 2012). Inhibiting the predicted iEGA TF, Mycn, disrupted embryo morphology, impeded cytokinesis, blocked early development and disrupted iEGA gene upregulation. Similarly, blocking the cancer-associated TFs, Erg or Atf4, which are also present in one-cell embryos and predicted to have iEGA-regulatory roles, also impeded preimplantation development (Asami et al., 2023). Thus, iEGA is a predictor of TFs that contribute to the onset of embryonic transcription, and which in many cases are regulatory oncogenes.

The number of iEGA genes (1,777 in mouse [FDR<5%], 1,322 in human [FDR<10%]) is clearly more than a handful (Ji et al., 2023), but in both mouse and human one-cell embryos, the amplitude of expression upregulation in iEGA was typically <2-fold and the mean log2 fold-change in mouse was 0.77 ± 0.03 (FDR<5%) (Asami et al., 2022; 2023). Increases were not population effects attributable to high-expressing outliers, as the analyses were on single cells (scRNA-seq). However, the induction of target genes by MYC is typically less than twofold (Baluapuri et al., 2020) and genes specific to ES cells undergo only modest increases during pluripotency induction (Chronis et al., 2017). The polycomb repressor is a pluripotency modulator that responds to low levels of transcription (Berrozpe et al., 2017), and a pleiotropic activator-repressor with a key gene-regulatory role in ES cells, CTCF, also exerts only a modest (less than 2-fold) effect on transcripts when CTCF and RAD21 are depleted (Narita et al., 2025). The nucleosome remodeling and deacetylation (NuRD) is essential for pluripotency, but eliminating its activity in ES cells results in gene expression changes of mostly less than twofold (Bornelöv et al., 2018; Miller et al., 2016). Modest gene expression level changes of less than twofold may thus be a hallmark of cellular potency regulation and transitions.

One-cell embryos represent the only obligate developmental node through which gamete-derived chromatin passes. Thus, iEGA may provide a unique read-out of chromatin marks transmitted from parents via their gametes with the potential to mediate epigenetic inheritance of acquired parental traits (Fitz-James and Cavalli, 2022; Asami et al., 2023).

## 4 Embryonic genome repression, EGR, and an iEGA ‘off’ switch

In addition to blocking iEGA, 10058-F4 treatment of mouse one-cell embryos caused upregulation of 923 genes (i.e., 61.5% of genes that were differentially expressed; FDR<5%) (Asami et al., 2023). This suggests that c-Myc either directly or indirectly represses gene expression in one-cell embryos (Perry et al., 2023). We refer to targeted suppression of transcription in the first 12 h after sperm-egg union as embryonic genome repression, EGR. It is possible that the disruption of EGR by 10058-F4 treatment contributes, at least partly, to acute developmental attenuation.

Some EGR genes become upregulated during preimplantation development (50 in mouse major EGA), indicating that for some, repression is transient. EGR pathways reflect downstream transitions from one-cell-stage to blastocyst development: lipid biosynthesis for the ∼26% plasma membrane area increase attending the first cell division (Duch et al., 2020; Pratt, 1990), maternal factor catabolism (Wu and Dean, 2020), and the downstream metabolic transition from oxidative phosphorylation to glycolysis (Redel et al., 2012).

It is established that c-Myc can behave as a transcriptional repressor (Grandori et al., 2000; Wanzel et al., 2003) and may thus act as a poised transcriptional (co)repressor that switches to or from (co)activator-mode, raising the possibility that c-Myc is differentially regulated to perform each function simultaneously at different loci in one-cell embryos. Like c-Myc, other transcriptional regulators predicted by iEGA have documented activator and repressor functions, including Atf4, Erg, Mycn, E2F1, Mitf, c-Rel and Foxm1 (Asami et al., 2023), but it is unknown whether they contribute to EGR. Our understanding about these activator-repressors largely derives from adult disease, but their principal normative physiological roles may include regulating early development.

Expression of most (61.6%) mouse iEGA genes had markedly declined by the two-cell stage (Asami et al., 2023). In the mouse, this coincides with major EGA, in which there is a relatively high-amplitude transcriptional upregulation of a larger set of genes (see below) (Hamatani et al., 2004). This situation is mirrored by human embryos when allowance is made for the later occurrence of human major EGA at the eight-cell stage: human one-cell embryo (iEGA) transcript levels remained elevated until around the eight-cell stage, when they declined (Asami et al., 2022). It thus appears iEGA is switched off as major EGA is initiated in human and mouse embryos.

## 5 Waves of early embryonic transcription

The dynamics of mouse EGA include iEGA (which segues to minor EGA) in one-cell embryos, followed by major EGA at the two-cell stage (Hamatani et al., 2004; Park et al., 2013; Wang et al., 2004; Xue et al., 2013; Zeng and Schultz, 2005). Mouse iEGA is refractory to the Pol II inhibitor, α-amanitin (Asami et al., 2023), which is a feature shared with minor EGA (Hamatani et al., 2004; Zeng and Schultz, 2005). In minor EGA, as in genome activation in one-cell embryos following nuclear transfer, the carboxy-terminal domain of the PolII large catalytic subunit, Rpb1, is hypophosphorylated (Bellier et al., 1997; Miyamoto et al., 2018). Rpb1 is canonically phosphorylated in active PolII and indeed becomes phosphorylated in major EGA (Bellier et al., 1997), so iEGA seems to be mediated by a distinctive PolII mechanism. These findings are consistent with iEGA and minor EGA employing related and idiosyncratic transcriptional mechanisms, and support the idea that they describe the same transcriptional phase.

By contrast, major EGA constitutes a second transcriptional wave, rather than a simple continuation of iEGA (Asami et al., 2023). In this model, iEGA is initiated by maternal factors immediately after fertilization, but largely yields to a new program of higher-amplitude expression driven by distinctive TFs in major EGA (Figure 1). Proposed modulators of major EGA include Ccnt2 (Zhang et al., 2022), Dppa2 (Eckersley-Maslin et al., 2019), Dppa4 (Guo et al., 2024), DUXBL (Vega-Sendino et al., 2024), Kdm1a (Ancelin et al., 2016), Klf17 (Hu et al., 2024), MLX (Wang et al., 2022), NAT10 (Cui et al., 2025), Nr5a2 (Gassler et al., 2022), Nuclear transcription factor Y subunit-α, NFYA (Lu et al., 2016), Obox (Ji et al., 2023), PRDM10 (Seah et al., 2025), Rfx1 (Wang et al., 2022), Smarcd2 (Zhang et al., 2022), Tprx1 (Zou et al., 2022), Yap1 (Yu et al., 2016) and YY1 (Wang et al., 2024). Of these, *Obox5* (FDR, 2.08E-26), *Yap1* (FDR, 1.65E-04), *Ccnt2* (FDR, 3.42E-03) and *Klf17* (FDR, 6.69E-22) are upregulated in iEGA (Figure 1). Treatment of mouse one-cell embryos with the protein synthesis inhibitor, cycloheximide, disrupts major EGA (i.e., at the two-cell stage) and development (Hu et al., 2024), suggesting that the translation of canonically-spliced transcripts produced during iEGA contributes to the transcriptional circuitry required for major EGA. Indeed, genes for several putative major EGA regulators (including orphan receptor genes, but not *Nr5a2*) are themselves upregulated in iEGA, suggesting that iEGA primes major EGA (Figure 1). We now describe selected examples of putative major EGA regulators.

Obox. The PRD-like homeobox domain transcription factor family, Obox, apparently regulates major EGA, as mice deficient for maternally-transcribed *Obox1*-*5* and *Obox7* expressed at the one-cell stage underwent impaired transcription and two-to-four-cell arrest (Ji et al., 2023). Activation by Obox family members is thought to exhibit redundancy and involves depositing H3K27ac at GC-poor promoter and enhancer regions, opening chromatin and pre-configuring PolII (Liu et al., 2020). This redundancy may involve the related TF, Tprx1 (Zou et al., 2022).

Nr5a2. The orphan nuclear receptor, Nr5a2, has been shown to upregulate major EGA genes in mouse two-cell embryos and to be required for progression beyond the two-cell stage (Gassler et al., 2022). Nr5a2 promotes chromatin accessibility and binds to motifs within short interspersed nuclear element (SINE) B1 (B1-elements) in the mouse, and in human counterparts, *Alu* retrotransposons, present in *cis*-regulatory regions of major EGA genes (Gassler et al., 2022). Chemical inhibition of Nr5a2 resulted in significant (FDR<5%) under-expression of 535 genes compared to controls (Gassler et al., 2022). However, genetic removal of maternal Nr5a2 produced the conclusion that Nr5a2 is dispensable for major EGA and that Nr5a2 may cooperate with other developmental TFs, such as Krüppel-like factors (below), to establish robust chromatin accessibility (Festuccia et al., 2024). In addition, Smart-seq analysis has suggested that *Nr5a2* is activated after major EGA (Oomen et al., 2025). The evolutionary processes that gave rise to B1/Alu-mediated major EGA also require explanation. B1-elements and *Alu*-repeats evolved from 7SL RNA and are mostly found in introns and upstream gene regulatory elements (Tsirigos and Rigoutsos, 2009): open chromatin at major EGA genes may thus not be the result of retroviral integration, but its cause. Integration at hundreds or thousands of loci associated with major EGA presumably occurred independently in mouse and human genomes to yield a selective advantage (or at least, not a disadvantage), but transcriptional activation must clearly have been successful before retroposition took place. The roles of Nr5a2 and retrotransposons in major EGA are therefore unclear. Genes encoding nuclear receptor family members Nr3c1, Nr2e1 and Nr6a1 are upregulated in iEGA, whereas Nr5a2 is neither upregulated in, nor a predicted regulator of iEGA (Asami et al., 2023). There is little evidence that Nr5a2 functions as a pioneer factor for iEGA.

Klf17. Krüppel-like factor 17 (Klf17) involvement in mouse and human major EGA has recently been inferred from genetic and proteomic analyses, and it may mediate PolII pre-configuration at the early two-cell stage (Taubenschmid-Stowers et al., 2022; Hu et al., 2024).

Yap1. Yes-associated protein 1 (Yap1) is highly expressed in mouse and human oocytes and early embryos. Maternally-derived Yap1 is necessary for major EGA, and Yap1 gradually translocates from the cytoplasm to the nucleus during early development (Yu et al., 2016). The *Yap1* gene is expressed in mouse iEGA 6 h post-fertilization, and it is a predicted upstream regulator of iEGA genes including *Rrm2*, *Cdc25a* and *Pdcl* whose expression increases after 10 h (Asami et al., 2023).

## 6 Relationship of EGA/EGR to embryoids

The genesis *in vitro* of embryoids (e.g., blastoids) from naïve PSCs skips multiple embryonic processes that follow fertilization (Boiani, 2024; Kagawa et al., 2022; Rivron et al., 2018). Among these processes are iEGA, major EGA and the downregulation of iEGA transcripts (EGR) that more-or-less coincides with major EGA (Asami et al., 2022; 2023; Perry et al., 2023). The expression profiles of genes undergoing regulation during EGA and EGR may be important for key down-stream developmental events, but they are manifest before the establishment of naïve pluripotency in blastocyst-stage embryos. Thus, even though the intracellular history of iEGA expression may be critical to mapping the developmental trajectory of embryoids, it may be difficult to trace back from later developmental stage embryos (e.g., blastocysts) or other entities from which naïve PSCs are derived. This concept potentially undermines confidence about the downstream - perhaps far-downstream - developmental potential of systems derived from naïve PSCs.

## 7 Concluding comments

We suggest that two waves of embryonic transcription follow fertilization in early preimplantation development: iEGA and major EGA. Both share conserved features with their respective counterparts in mouse and human. iEGA reflects the initiation of transcription in one-cell embryos, and minor EGA is a continuation of it. The second gene expression wave (major EGA) involves both a boost in transcriptional amplitude and qualitative differences compared to iEGA. Blocking iEGA can precipitate acute developmental arrest (Asami et al., 2023), suggesting that it is of critical functional importance. Cell cycle pathways feature in mouse and human iEGA, and may play roles in the intricate and anomalous choreography between cell volume and cell cycle length in early embryos. Moreover, iEGA may prime major EGA, as genes for several candidate regulators of major EGA are upregulated in iEGA. This has mechanistic implications and suggests that iEGA holds clues to the identities of major EGA regulators. Similarly, iEGA may illuminate chromatin alterations in mouse and human gametes that reflect parentally-acquired traits (e.g., obesogenic traits): epigenetic inheritance (Fitz-James and Cavalli, 2022). Thus, iEGA may be both of functional importance in its own right, and report additional key steps in the early embryo.

## Data Availability

The original contributions presented in the study are included in the article/supplementary material. Further inquiries may be directed to the authors.
